# New and Non-Official Remedies

**Published:** 1912-02

**Authors:** 


### New and Non-Official Remedies.

(Begun in October, 1911, issue.)

### QUININE ANALOGUES.

(Aristochin), Quinine carbonate. Slowly acted on by acids, hence less gastric disturbance. Same dose as quinine.

(Chinaphenin), Quinine and Phenitidin carbonate. Tasteless, water-insoluble, slowly dissolved in acids. Combines the actions of quinine and phenacetin. Same or slightly greater dose than quinine.

(Euquinine), Quinine and Ethyl carbonate. Practically same advantages and effects as aristochin.

Quinine lygosinate. Styptic and antiseptic, used in dusting powder, incorporated into gauze, in suppository &c.

Quinine and urea hydrochloride. Used as quinine substitute, especially hypodermatically; also as cocaine substitute, ¼—1 per cent., injected into tissues; 10—20 per cent. topically.

(Salo-quinine), Quinine salicylate, tasteless, combining actions of quinine and salicylates. Salo-quinine salicylate, also tasteless, contains relatively more salicylic acid.

(Chinosol), normal Oxychinolin sulphate, is employed simply as an antiseptic, being feebly germicidal. Internally, 1-3 gram (5 grains) t.i.d.; externally 1:1000; in nose, vagina, bowel, etc. 1:5000; in eye 1:8000.

### VOLATILE OIL ANALOGUES.

(Carbosant), Santalyl carbonate. Oily, almost free from taste and odor. Similar to oil of sandal wood, in action and dose.

(Santyl), Santalyl salicylate, similar to above, said to pass stomach unchanged and to be split into constituents in intestine, hence less irritating and more gradual in action than sandalwood oil, and with combination of salicylate effect.

Menthyl ethyl glycolate (Coryfin). Oily, very slight peppermint odor, used internally and externally as menthol or oil of peppermint.

Oil of pine needles (from pinus pumilio). Inhalation, antiseptic, expectorant, rubefacient.

Oleoresin of parsley seed, contains Apiol, a parsley camphor, emmenogogue, etc.

(Oxaphor) camphor hydroxid, 50 per cent. in alcohol. Like camphor, respiratory sedative with no depression of heart or secretions.

Sodium cinnamate is scarcely analogous to oil of cinnamon, as it is said to produce cicatrization about inflammatory, especially tuberculous, foci, rather than to have a direct antiseptic and carminative effect. Dose 1 milligram, increased gradually to 2 centigrams, intravenously, three times weekly.

### ICHTHYOL ANALOGUES.

Ichthyol, Ammonium ichthyol, Ammonium sulpho-ichthyolate (exact composition and constitution in doubt) is claimed to be vaso-constrictor, antiseptic and "alterative." Internally and locally as in vaginal, rectal suppositories, urethral bougies, 2—20 centigrams (1-3—3 grains). As injection, 1—3 per cent.

Calcium ichthyol, Calcium sulpho-ichthyolate, brown, tasteless, water-insoluble powder.

(Ichthalbin), Albumin sulpho-ichthyolate, greyish white, tasteless powder, passing stomach unchanged, rendered soluble in intestine. Same dose as above.

(Ichthargan), Silver sulpho-ichthyolate, see under Silver.

(Ichthermol), Mercury sulpho-ichthyolate, see under Mercury.

Ichthoform), Ichthyol formaldehyde.

(Ferrichthyol), Ferric sulpho-ichthyolate, see under Iron.

Lithium ichthyol, Lithium sulpho-ichthyolate.

Sodium ichthyol, Sodium sulpho-ichthyolate.

Tumenol (venale) a mixture of tumenol sulphone and tumenol sulphonic acid, a dark, oily substance, insoluble in water, combining with fats and used mainly in ointments, plasters, glycerin-water emulsions, 5—20 per cent.

(Tumenol-ammonium), Ammonium tumenol-sulphonate, water soluble, also combined as above, for which it is a purer substitute.

All of these products of fossil organic matter are of somewhat mixed composition, the chemic nomenclature is not established nor have the physiologic actions been thoroughly worked out.

